# An Invasive Mammal (the Gray Squirrel, Sciurus carolinensis) Commonly Hosts Diverse and Atypical Genotypes of the Zoonotic Pathogen Borrelia burgdorferi Sensu Lato

**DOI:** 10.1128/AEM.00109-15

**Published:** 2015-06-04

**Authors:** Caroline Millins, Agnieszka Magierecka, Lucy Gilbert, Alissa Edoff, Amelia Brereton, Elizabeth Kilbride, Matt Denwood, Richard Birtles, Roman Biek

**Affiliations:** a Institute of Biodiversity, Animal Health and Comparative Medicine, University of Glasgow, Glasgow, Scotland; b The Boyd Orr Centre for Population and Ecosystem Health, University of Glasgow, Glasgow, Scotland; c James Hutton Institute, Craigiebuckler, Aberdeen, Scotland; d School of Veterinary Medicine, University of Glasgow, Glasgow, Scotland; e Institute of Biological and Environmental Sciences, University of Aberdeen, Aberdeen, Scotland; f Faculty of Health and Medical Sciences, University of Copenhagen, Copenhagen, Denmark; g School of Environment and Life Sciences, University of Salford, Salford, England

## Abstract

Invasive vertebrate species can act as hosts for endemic pathogens and may alter pathogen community composition and dynamics. For the zoonotic pathogen Borrelia burgdorferi sensu lato, the agent of Lyme borreliosis, recent work shows invasive rodent species can be of high epidemiological importance and may support host-specific strains. This study examined the role of gray squirrels (Sciurus carolinensis) (*n* = 679), an invasive species in the United Kingdom, as B. burgdorferi sensu lato hosts. We found that gray squirrels were frequently infested with Ixodes ricinus, the main vector of B. burgdorferi sensu lato in the United Kingdom, and 11.9% were infected with B. burgdorferi sensu lato. All four genospecies that occur in the United Kingdom were detected in gray squirrels, and unexpectedly, the bird-associated genospecies Borrelia garinii was most common. The second most frequent infection was with Borrelia afzelii. Genotyping of B. garinii and B. afzelii produced no evidence for strains associated with gray squirrels. Generalized linear mixed models (GLMM) identified tick infestation and date of capture as significant factors associated with B. burgdorferi sensu lato infection in gray squirrels, with infection elevated in early summer in squirrels infested with ticks. Invasive gray squirrels appear to become infected with locally circulating strains of B. burgdorferi sensu lato, and further studies are required to determine their role in community disease dynamics. Our findings highlight the fact that the role of introduced host species in B. burgdorferi sensu lato epidemiology can be highly variable and thus difficult to predict.

## INTRODUCTION

The introduction of an invasive species can pose threats to human health and biodiversity by introducing novel pathogens or if the species acts as a host for endemic pathogens preexisting in the native wildlife population (1). This may allow pathogen persistence in new areas and facilitate pathogen spread as the invasive host expands its range. The introduced host may act as a maintenance host alone or become part of a maintenance community (2) with native hosts.

In the ecology of Lyme borreliosis, a tick-borne zoonosis, the host community composition is of critical importance in determining the proportion of infected ticks in the environment, a key risk factor for human infections (3–5). An invasive species can act as an alternative host for endemic pathogens in a type of “spillover” effect, with subsequent “spill back” of the pathogen to native hosts (6, 7). Either an overall amplification or dilution effect on disease dynamics may be seen following the addition of an invasive species, depending on how it affects the host community capacity (7). For example, an amplification effect was found following the introduction of the Siberian chipmunk (Tamias sibericus), an invasive rodent species, to forests in France. Invasive Siberian chipmunks in France have been found to host multiple species of Borrelia burgdorferi sensu lato, are frequently parasitized by Ixodes ricinus, and contribute more to Lyme borreliosis risk than do native rodents (8–10).

Lyme borreliosis is caused by spirochete bacteria in the B. burgdorferi sensu lato species group and is among the most significant vector-borne zoonoses in the Northern Hemisphere (11, 12). The B. burgdorferi sensu lato species group includes multihost pathogens with both generalist and specialist strategies (13). B. burgdorferi sensu lato is transmitted to wildlife hosts by generalist ticks from the Ixodes persulcatus species group, and over 100 animal species have been identified as hosts, including rodents, birds, insectivores, carnivores, and reptiles (13). Infection within a competent host produces a chronic systemic infection that is transmitted horizontally to blood-feeding ticks (14). Transmission by cofeeding (15, 16) and transovarial (vertical) transmission (17) are thought to be less significant epidemiologically.

The relative contributions of reservoir host species to B. burgdorferi sensu lato transmission dynamics in ecological studies are often assessed by measuring the proportion of infected individuals and quantifying tick burdens. Typically, ear punch biopsies are used to determine host infection prevalence, considered to represent disseminated, chronic infections. As the level of bacteremia in infected animals can fluctuate, this has been shown to be a more sensitive method than testing blood (18) and allows longitudinal ecological surveys to be performed without disrupting the host community.

Of the 19 described genospecies of B. burgdorferi sensu lato (13, 19), four have been reported in the United Kingdom (20, 21).They are the host specialist genospecies Borrelia afzelii, which is maintained by rodent hosts (22), and Borrelia garinii and Borrelia valaisiana, which are maintained by birds (23–25), and the generalist genospecies B. burgdorferi sensu stricto, which is maintained by both rodents and birds (26, 27). Multiple species and strains of the bacteria can circulate in a single geographic location (23, 28) and are thought to be maintained by multiple niche polymorphisms, with different hosts providing different niches, and by negative frequency-dependent selection (29). Laboratory studies have confirmed that B. burgdorferi sensu lato strains have varying fitness in different hosts (30). More recently, the host specificity of certain genotypes within B. burgdorferi sensu lato genospecies has been described in invasive Siberian chipmunks and native bank voles from the same forest site in France (31).

The gray squirrel (Sciurus carolinensis) is an invasive, nonnative species in the United Kingdom and is regarded as a pest species under national legislation. In their native range in eastern North America, they are known to be a competent host of B. burgdorferi sensu stricto (3). Following multiple introductions from 1876 to 1929 (32), gray squirrels have become widely established in the United Kingdom, are linked to the decline of the native red squirrel (Sciurus vulgaris) (33, 34), and cause widespread damage to forests (32). The population is estimated at over 2 million, with at least 200,000 gray squirrels present in Scotland (35). They are common in habitats used frequently by people, such as urban parks and suburban areas of the United Kingdom, and thus are a potentially important risk factor for human exposure to B. burgdorferi sensu lato. There is limited knowledge of how invasive gray squirrels contribute to B. burgdorferi sensu lato dynamics in the United Kingdom, which genospecies they can transmit, and the environmental and host risk factors for infection. A study from one woodland in the south of England found that gray squirrels were frequently parasitized with I. ricinus larvae and nymphs (36). Experimental infection of two gray squirrels and testing of larvae that had fed on one naturally infected gray squirrel (xenodiagnosis) has shown them to be competent hosts for the rodent-associated genospecies B. afzelii (37).

This study aimed to evaluate the role of gray squirrels as hosts of B. burgdorferi sensu lato in the United Kingdom by (i) quantifying the prevalence of host infection, tick burdens, and infection of larvae found on squirrels; (ii) genotyping B. burgdorferi sensu lato infections detected in gray squirrels and questing I. ricinus nymphs to determine if certain genotypes are associated with gray squirrels; (iii) quantifying the frequency of disseminated infections in gray squirrels and determining whether they consistently correspond to positive ear biopsy results; and (iv) identifying the environmental risk factors and host characteristics associated with B. burgdorferi sensu lato infection in gray squirrels.

## MATERIALS AND METHODS

### Gray squirrel sampling.

**FIG 1:**
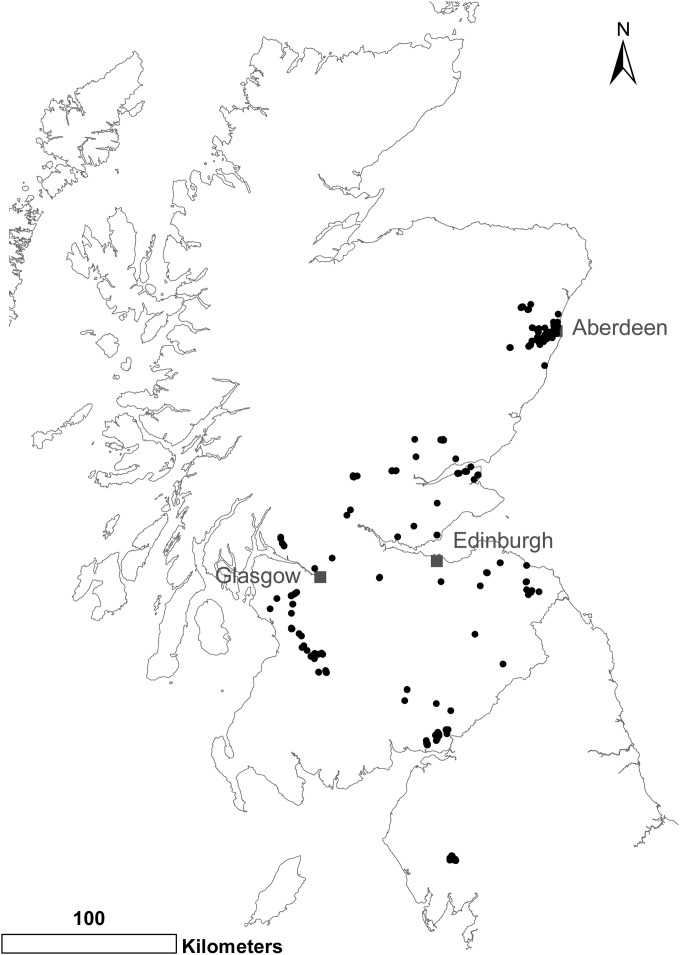
Map of gray squirrel (S. carolinensis) sampling locations in Scotland and northern England. A total of 679 gray squirrels were sampled in 2012 and 2013. Created in ArcMap 10 (ESRI). Great Britain and Scotland outlines © Crown Copyright and Database Right 2011. Ordnance Survey (Digimap License).

Gray squirrel carcasses and samples were provided by conservation groups carrying out gray squirrel population control in Scotland and northern England. Gray squirrels were live trapped and humanely killed by trained personnel in accordance with relevant United Kingdom legislation. A total of 679 squirrels were sampled from 9 regions carrying out gray squirrel control (range, 22 to 292 squirrels per region; median, 50) (Fig. 1; see Fig. S4 in the supplemental material). The squirrels were trapped in 2012 and 2013 during the tick questing period of March to October. To investigate temporal variation in B. burgdorferi sensu lato prevalence, at least 30 squirrels were collected each month from one region (the northeast; *n* = 292) from March to October, as well as a total of 31 individuals from the winter months (November to February). The gray squirrel carcasses were placed in sealed plastic bags immediately after euthanasia and frozen at −20°C. The date and GPS location of capture were recorded. The carcasses were later defrosted in the laboratory, and a standardized postmortem and tissue-sampling protocol was carried out. During this procedure, the mass (in grams), body length (nose to anus in millimeters), tarsus length (in millimeters), and sex were determined. Retroperitoneal fat (kidney fat) stores were graded as either present (good) or absent/minimal (poor). The age class was determined based on body weight; gray squirrels were classified as juveniles (<200 g), subadults (200 to 500 g), and adults (500 g) (38). Previous studies using incremental lines laid down in tooth cementum (39) have shown that 97% of squirrels weighing <500 g are subadults (40). Samples of the pinnae, heart, spleen, kidney, and bladder were frozen at −20°C for subsequent DNA extraction. Freezers for storing entire carcasses were not available in some locations, and instead, samples of ear pinnae were collected (*n* = 67) by the control officer; the samples were preserved in 90% ethanol and submitted, together with the date, trap location, mass, and sex of the squirrel.

### Tick quantification.

As the majority of ticks on squirrels are thought to attach on the head and ears (36), these areas were carefully examined, and any attached ticks were removed. Ticks were also removed from the rest of the carcass by brushing the coat; the ventral, dorsal, and lateral surfaces were brushed 10 times onto a white tray (41). The bag the squirrel was stored in was also carefully examined for detached ticks, using a magnifying glass where necessary. All ticks were removed, counted, and preserved in 70% ethanol and later identified to species and life stage using a light microscope and standard keys (42; http://bristoltickid.blogs.ilrt.org/).

### DNA extraction, B. burgdorferi sensu lato PCR, and genospecies determination.

DNA extractions were carried out in a dedicated laboratory space separate from areas where PCR products were handled. The DNA extractions were carried out on 25 mg of ear tissue from each squirrel using DNeasy blood and tissue kits (Qiagen) according to the manufacturer's instructions. When tick larvae were found on squirrel carcasses, DNA extractions were carried out on a pooled sample of up to 10 larvae from each squirrel. Larval ticks were finely minced with scissors prior to extraction with DNeasy blood and tissue kits using the Qiagen supplementary protocol for the purification of DNA from ticks to detect B. burgdorferi sensu lato (43). A negative extraction control was included with every 23 samples. A nested PCR that targets the 5S-23S rRNA intergenic spacer (IGS) region of B. burgdorferi sensu lato was carried out on DNA extracts from ear- and host-derived larval ticks, as described previously (44). Detection of the 225-bp product was by electrophoresis of PCR products on a 2% ethidium bromide gel; the genospecies of B. burgdorferi sensu lato was determined by Sanger sequencing of the product in the forward and reverse directions at the DNA and Sequencing Service, University of Dundee. A negative PCR control and a positive control (Borrelia lusitaniae, a genospecies not found in the United Kingdom) were included in each PCR run.

To determine the genospecies, each sequenced and trimmed IGS PCR product was subjected to a BLAST search against the National Center for Biotechnology Information (NCBI) nucleotide BLAST database. To confirm the genospecies identity, each sequence was further examined for several polymorphisms within the IGS region that discriminate between the different genospecies (Table 1; see Fig. S5 in the supplemental material). These discriminatory sites were found by selecting 100 representative sequences for each of the four genospecies found in the United Kingdom from the NCBI nucleotide BLAST database. An alignment was made in Geneious version 7.0.6 (Biomatters Ltd.), and the sequences were examined for discriminatory sites. If different discriminatory sites in a single sequence were classified as different genospecies, the sample was classed as a mixed-genospecies infection.

**TABLE 1:** Test sites for discriminant analysis within the 5S-23S rRNA intergenic spacer region used to determine the genospecies for B. burgdorferi *sensu lato*

Genospecies	Site sequence for test no.^*a*^:					
	1	2	3	4	5	6
B. afzellii	**TTAAA**	T		**AAAACA**	**AAT**—	G
	TA-AA			GTTTTA	AAA—	
B. garinii	**TATG-**	**G**	GTTTATG	**GTTTTA**	AAA—	G
	TATAT	T	GTTCATT	AAAACA	AAAA-T	
			GTTCATG		AAAA–	
			ATTTATG			
B. burgdorferi sensu stricto	AT-GT	A	ATTGGTG	**GGGTTG**	AAAA-T	C
				GTGTTG	AAA—	T
B. valaisiana	TATAT	A	GTTCATG	**GTTTTA**	AAA—	**G**
	TATGT		GTTTATG	ATTTAA	AAAA-T	T
	T—-		ATTCATG			
	–TAT					

a Figure S5 in the supplemental material shows the positions of the test sites. Variation at test sites is from 100 randomly selected sequences of each genospecies selected from the NCBI database (more common sequence polymorphisms are in boldface).

### Genotyping B. burgdorferi sensu lato detected in gray squirrels and questing nymphs.

To test whether certain B. burgdorferi sensu lato genotypes were associated with gray squirrels, we compared genotypes detected in gray squirrels to those detected in the questing I. ricinus population in Scotland. All gray squirrel samples that tested PCR positive for B. burgdorferi sensu lato, as well as questing I. ricinus nymphs from a separate survey (described below), were sequenced at two gene loci. The two gene loci are expected to have different phylogenies and selective pressures. One is a conserved housekeeping gene (*clpA*) located on the main chromosome, and the second is a highly variable infection-related gene (*ospC*) located on a plasmid, which has been used to document host specificity in previous studies (31, 45).

The first locus is a 579-bp *clpA* fragment from a widely used multilocus sequence typing (MLST) scheme based on eight conserved, chromosomally located housekeeping genes (46). The amplification protocols were as previously described (46), and the PCR products were sequenced in both forward and reverse directions using Sanger sequencing. Consensus sequences of forward and reverse sequences were made using the Geneious (Biomatters, Ltd.) alignment tool. They were aligned with known *clpA* sequences from the MLST database (http://borrelia.mlst.net/) and trimmed to the appropriate length. The allele identity was found by comparing the trimmed consensus sequence to the B. burgdorferi sensu lato MLST database. Samples were also tested at the *ospC* locus, using a nested-PCR protocol that produced an approximately 500-bp *ospC* fragment (47). Any samples that failed to amplify were retested using an alternate primer set for the same *ospC* fragment (31). The *ospC* products were aligned and trimmed to the same length (472 to 484 bp), and all distinguishable alleles were determined.

In order to compare *clpA* genotypes found in squirrels to those found in the tick population in Scotland, data from a previous study that tested over 3,600 questing nymphs from 25 locations across Scotland were used (48). The blanket-dragging method to collect questing nymphs from vegetation, DNA extraction, and PCR methods have been previously described (20). To measure *ospC* diversity in Scotland, *ospC* data were generated from B. burgdorferi sensu lato-infected questing nymphs collected in a separate study in Scotland (C. Millins, unpublished data). Briefly, this study collected over 5,000 questing nymphs from 27 locations across Scotland using blanket dragging. The nymphs were stored in 70% ethanol and individually extracted using an alkaline hydrolysis method with 0.7 M ammonium hydroxide (49). They were tested for B. burgdorferi sensu lato infection using a real-time PCR method described previously (50).

Due to the large number of reported B. garinii clpA alleles (51), the sample size needs to be taken into account when comparing the diversity of B. garinii *clpA* alleles found in squirrels in this study to those reported from questing ticks. For this, rarefaction analyses were performed in the R package vegan (52) (see Fig. S1 in the supplemental material). Separate rarefaction curves were estimated for questing ticks from Europe and England using data on *clpA* alleles and their reported frequencies from the B. burgdorferi sensu lato MLST database (http://borrelia.mlst.net/) and from Scotland (this study and reference 48).

### Phylogenetic analysis to detect potential host specificity.

A phylogeny-trait correlation analysis was carried out to investigate if there were gray-squirrel-specific genotypes of B. burgdorferi sensu lato (53). This approach can be used to quantify how phenotypic characteristics of a pathogen, such as the host species or the location it was sampled from, are correlated with shared ancestry, as represented by a phylogenetic tree of the pathogen. Maximum-likelihood trees based on alleles from squirrels and questing ticks from Scotland were estimated for each of the two loci, *clpA* and *ospC*, in MEGA version 6 (54) using optimal substitution models for each data set. Separate trees were generated for the two most frequent genospecies found to be infecting gray squirrels in Scotland, B. garinii and B. afzelii. The program Mr Bayes 3.2.2 (55) was used to generate a posterior distribution of phylogenetic trees using Bayesian inference and the nucleotide substitution models previously selected in MEGA 6. Alleles recorded more than once were included multiple times, reflecting the frequency at which they were observed. For each data set, 1,000 trees were sampled from the posterior distribution following convergence. Based on these trees, we tested for evidence of phylogenetic clustering using the program BaTS 1.0 (53). We tested the null hypothesis that B. burgdorferi sensu lato *clpA* and *ospC* alleles detected in gray squirrels are a random subset of those found in questing ticks.

### Detecting disseminated infections with B. burgdorferi sensu lato.

Separate DNA extractions were carried out on heart, spleen, kidney, and bladder samples from 20 squirrels with PCR-positive ear biopsies and 20 with PCR-negative ear biopsies, both groups selected randomly. These tissues were selected because they are organs known to become infected in other rodent species (18, 56). The DNA extraction protocol described above was followed, and 25 mg of all tissues apart from the spleen (10 mg) was sampled for the extraction, according to the manufacturer's instructions. All organ DNA extracts were tested by the IGS PCR method for B. burgdorferi sensu lato (44) as described above.

### Environmental and host predictors of B. burgdorferi sensu lato infection.

Analysis of environmental variables was conducted in ArcMap 10.0 (ESRI, Redlands, CA [2010]). The woodland type at the squirrel trap site was obtained by performing a spatial join between the squirrel trap location and a Forestry Commission polygon shape file containing information on woodlands of greater than 0.5 ha (National Forest Inventory Great Britain 2012 shapefile [available at http://www.forestry.gov.uk/datadownload]). Based on the accuracy of trap location records (96% of the trap sites were recorded with an accuracy of 100 m or less) and a conservative estimate of the squirrel home range of 150 m (57), the woodland type was that attributed to the area in which the trap site fell or within 150 m of the trap site. Only two forest types were considered suitable habitat for gray squirrels, as they contained mature trees (58): broadleaf (50 to 100% broadleaf tree cover) and coniferous (50 to 100% conifer tree cover).

All statistical analyses were carried out in R version 3.1 (R Development Core Team, Vienna, Austria) using the lme4 package (59) for generalized linear mixed models. A binomial model was used to examine the relationship between the B. burgdorferi sensu lato infection status (infected or not infected) as the outcome variable; the explanatory variables age, sex, kidney fat score, tick presence or absence, woodland type, easting and northing of the capture site, and capture date as an annual sine wave; and woodland site as a random effect to control for spatial pseudoreplication. Easting and northing and their interaction were included to test for the possible presence of a spatial gradient in B. burgdorferi sensu lato infection. Woodland type was included because deciduous woodland in Scotland has previously been found to have significantly higher numbers of infected questing nymphs, and this may be a useful proxy for the presence of other competent transmission hosts, such as small mammals and birds (20). All three tick life stages were included in the tick presence variable. Although larvae are rarely infected with B. burgdorferi sensu lato, the more numerous larval burdens were found to be significantly correlated with nymphal burdens and thus act as a proxy for exposure to B. burgdorferi sensu lato (Wilcoxon signed rank test; *P* = 0.001).

A maximal global model was fitted that included all main effects and a single interaction term between northing and easting. Model selection was based on Akaike's information criterion (AIC) and followed a backward stepwise model selection approach. Variables were dropped sequentially by evaluating the effects of their removal on the model's AIC (60), and the model with the lowest AIC value was selected.

### Nucleotide sequence accession numbers.

The nucleotide sequence data for *ospC* generated in this study are available in NCBI GenBank under accession numbers KP644249 to KP644308.

## RESULTS

A total of 311 (45.8%) female and 325 (47.9%) male gray squirrels were received; sex was not recorded for 43 (6.3%) individuals. A total of 525 (77.3%) squirrels were adults, 148 (21.8%) were subadults, 5 (0.7%) were juveniles, and 1 individual (0.2%) did not have an age recorded. The woodland type at or within 150 m of the trap site was broadleaf at 493 (72.6%) trap sites and coniferous at 153 (22.6%). The woodland type was not recorded for 33 (4.9%) squirrels, as there was no recorded woodland within 150 m of the trap site.

### Tick burdens.

Tick burdens were quantified for 579 (85%) squirrels. A total of 240 (41.5%) squirrels carried one or more ticks. All ticks (*n* = 1,585) removed from squirrel carcasses were identified as I. ricinus; 1,120 (77%) ticks were larvae, 361 (22.8%) were nymphs, and 2 (0.1%) were adult females. Among squirrels carrying one or more ticks at any life stage, the median number of larvae was 2 (range, 0 to 117), and the median number of nymphs was 1 (range, 0 to 37).

### B. burgdorferi sensu lato infection.

The overall prevalence of B. burgdorferi sensu lato infection in gray squirrels based on PCR testing of ear biopsy specimens was 11.9% (95% confidence interval [CI], 9.7% to 14.6%). All four genospecies present in the United Kingdom were detected. B. garinii was the most prevalent genospecies and was found in 51 (63%) infected squirrels, followed by B. afzelii in 20 (24.7%), B. valaisiana in 3 (3.7%), and B. burgdorferi sensu stricto in 2 (2.5%) infected squirrels. Mixed infections with two genospecies were detected in three (3.7%) infected squirrels. Two mixed infections were with B. garinii and B. burgdorferi sensu stricto, and one was with B. garinii and B. afzelii. In addition, coinfections were detected in two squirrels based on B. garinii and B. afzelii detection in different tissue samples (skin and bladder). A genospecies could not be determined in two infected squirrels that tested positive by PCR.

Pooled larvae from 183 squirrels were tested. Testing of ear punch biopsy specimens from these individuals found that 21.3% were positive for B. burgdorferi sensu lato and 78.7% were negative. A total of seven (4%) of the larval pools (*n* = 183) tested PCR positive for B. burgdorferi sensu lato. Two of them came from squirrels that had a positive ear biopsy result for B. garinii, and larval pools also tested positive for B. garinii. The remaining five pools came from squirrels with PCR-negative ear biopsies. Three of these larval pools were positive for B. garinii, and two were positive for B. afzelii.

### Phylogenetic analysis to detect potential B. burgdorferi sensu lato host specificity.

**FIG 2:**
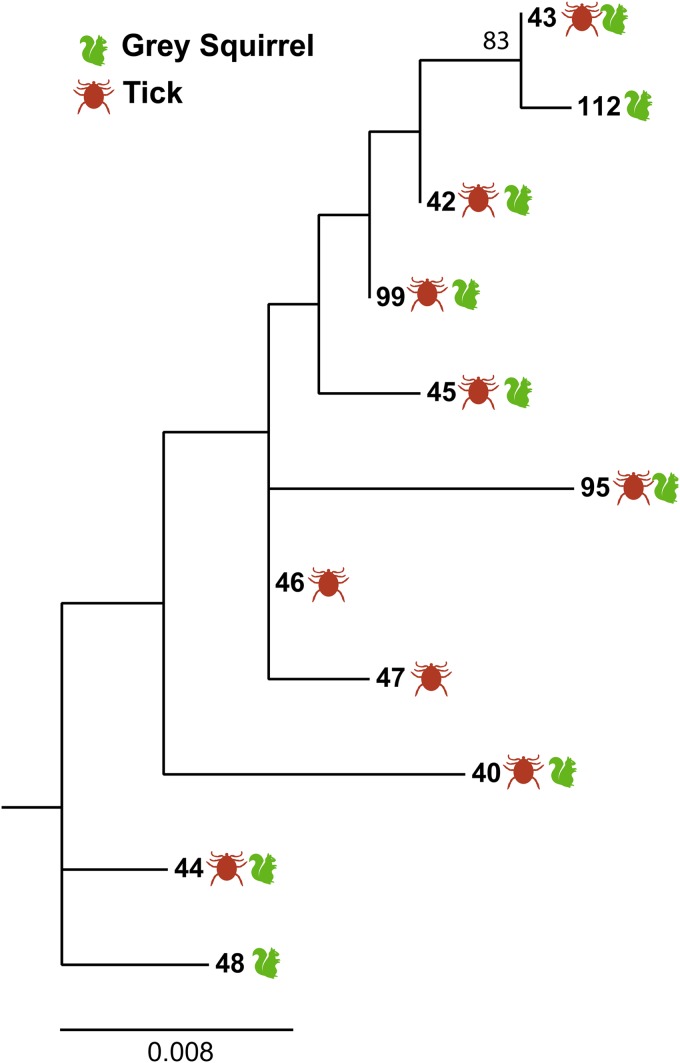
Maximum-likelihood (ML) phylogeny showing the diversity of *clpA* alleles from B. garinii originating from infected gray squirrels (S. carolinensis) (*n* = 20) and questing I. ricinus ticks (*n* = 30) from Scotland. B. burgdorferi sensu stricto (*clpA* allele 15) was used as an outgroup to root the tree (not shown). The allele numbers follow the classification from the B. burgdorferi MLST website (http://borrelia.mlst.net/). Nine alleles were detected in squirrels, alleles 40 (*n* = 3), 42 (*n* = 3), 43 (*n* = 4), 44 (*n* = 1), 45 (*n* = 2), 48 (*n* = 1), 95 (*n* = 2), 99 (*n* = 3), and 112 (*n* = 1). The ML tree was estimated based on the Hasegawa-Kishino-Yano model (78) in MEGA 6 (54), with 500 boot straps to evaluate branch support. Only bootstrap values greater than 70 are displayed on the tree. The scale bar represents nucleotide substitutions per site (genetic divergence).

A total of nine B. garinii *clpA* alleles, which were scattered widely across the entire tree of Scottish *clpA* alleles (Fig. 2), were detected in squirrels. All but two of these alleles had been previously recorded in questing ticks from Scotland (48). Alleles detected in squirrels did not show evidence of phylogenetic clustering relative to alleles found in questing ticks (Table 2). The trajectory of the rarefaction plots for *clpA* in ticks and squirrels (see Fig. S1 in the supplemental material) indicated that, while sampling of the diversity of *clpA* in the different populations was incomplete, a large part of the diversity had been captured.

**TABLE 2:** Testing for phylogenetic clustering of B. garinii *clpA* alleles by sampled species^*a*^

Statistic^*b*^	Observed mean (95% CI)	Null mean (95% CI)	*P* value
Association index	2.4 (1.5–3.2)	2.3 (1.9–2.7)	0.58
Parsimony score	16.1 (14–18)	15.9 (13.8–17.7)	0.52
Monophyletic clade size			
Gray squirrel	2.3 (1–3)	2.5 (2–4)	0.95
Tick	6.6 (6–8)	4 (2.9–6.2)	0.09

a See the maximum-likelihood tree shown in Fig. 2.

b The tests used three test statistics described previously (52). All three test statistics test the null hypothesis that the sampled species are distributed randomly among tip nodes. Association index, a measure of imbalance of internal nodes; parsimony score, the number of state changes in phylogeny; monophyletic clade size, a measure of the size of the clade sharing the same trait at the tips.

A total of 10 *ospC* alleles were detected in B. garinii-infected squirrels (see Fig. S2 in the supplemental material). These 10 alleles again represented much of the diversity seen in ticks sampled across Scotland. Although the phylogenetic cluster analysis detected some imbalance of internal nodes, indicating that some alleles were detected more commonly in one species than expected, there was no overall signal for clustering of squirrel *ospC* alleles relative to those detected in ticks (see Table S1 in the supplemental material), consistent with the results for *clpA*.

**FIG 3:**
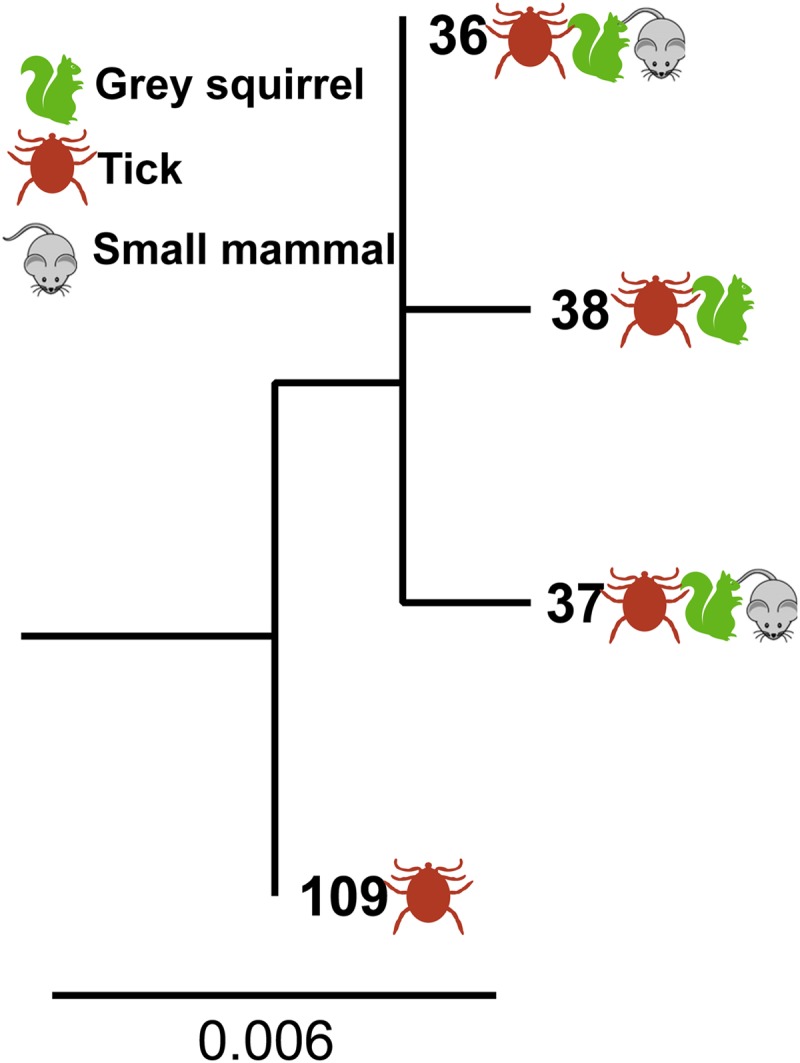
ML phylogeny of *clpA* alleles from B. afzelii in Scotland originating from infected squirrels (this study; *n* = 11), as well as small rodents (*n* = 11) and ticks (*n* = 32) sampled in a separate study (48). B. burgdorferi sensu stricto (*clpA* allele 15) is included as an outgroup (not shown). The numbering of alleles is according to that at the Borrelia MLST website (http://borrelia.mlst.net/). Three alleles were detected in gray squirrels, alleles 36 (*n* = 3), 37 (*n* = 4), and 38 (*n* = 4). The ML tree was estimated based on the Hasegawa-Kishino-Yano model (78) in MEGA 6 (54). The scale bar represents nucleotide substitutions per site (genetic divergence).

Three B. afzelii *clpA* alleles were detected in squirrels (Fig. 3). They included two *clpA* alleles previously reported from small mammals (bank voles [Myodes glareolus] and wood mice [Apodemus sylvaticus]) and questing ticks in Scotland (48), whereas the third had been previously reported in questing ticks (48). There was some evidence for alleles clustering by species based on significant results for two overall test statistics (see Table S2 in the supplemental material). However, the test statistic based on the size of the squirrel monophyletic clade was not statistically significant (*P* = 0.06). An additional phylogenetic cluster analysis was carried out to test whether there were spatial patterns in B. afzelii *clpA* allele distribution in gray squirrels. This was to investigate whether spatial patterns in B. afzelii genotypes as previously reported (51) could be driving clustering seen between species. This analysis found that *clpA* alleles from infected squirrels did cluster by the region the squirrels were trapped in (see Table S3 in the supplemental material).

Six *ospC* alleles were detected in B. afzelii-infected squirrels; four of these were also detected in questing ticks (see Fig. S3 in the supplemental material). No significant clustering of squirrel *ospC* alleles among those detected in ticks was found (see Table S4 in the supplemental material).

### Disseminated infection.

No evidence of disseminated infection with B. burgdorferi sensu lato was found in the heart, spleen, kidney, or bladder samples from 20 squirrels with a PCR-negative ear biopsy. An additional 100 spleen samples from randomly selected ear biopsy PCR-negative squirrels also tested PCR negative. Disseminated infection was found in 7 out of 20 squirrels that tested PCR positive on the ear biopsy: B. garinii was detected in the bladders of 2 squirrels, B. afzelii in the bladders of 2 squirrels, and B. garinii in the hearts of 3 squirrels. Two cases of mixed-strain infection with B. garinii were found, with different *clpA* and *ospC* alleles from the ear and bladder of the same squirrel. One case of mixed-strain infection with B. afzelii was found with different *ospC* alleles in the ear and bladder of the same squirrel.

### Host and environmental predictors of B. burgdorferi sensu lato infection.

**FIG 4:**
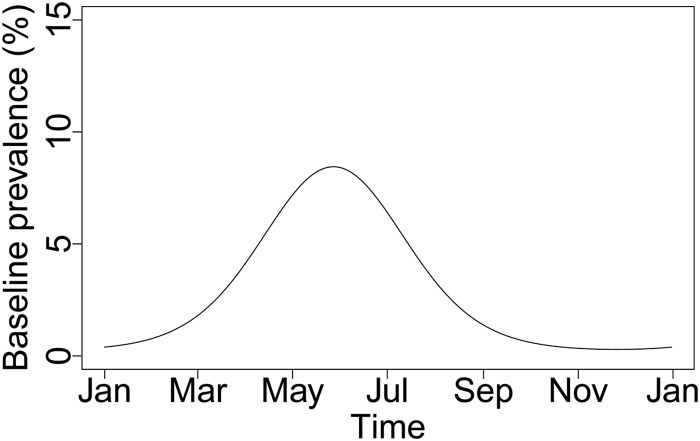
Predicted seasonal prevalence of B. burgdorferi sensu lato infection in gray squirrels from Scotland from the best-fit binomial generalized linear mixed model, which included the day (of the year) of capture, year, age, and tick presence, with a random effect of woodland. The depicted curve corresponds to adult gray squirrels sampled in 2012 without ticks.

Gray squirrels with data available for all variables were included in the binomial model for B. burgdorferi sensu lato infection (*n* = 515). The best-fit model included day (of the year) of capture, year, tick presence, and age, with a random effect of woodland (Table 3). Age improved the fit of the model slightly (ΔAIC [the change in AIC after removing each variable from the best-fit model] = 1.38), with subadults tending to be more likely to be infected (odds ratio [OR] = 1.83 [95% CI, 0.96 to 3.52]), though this parameter was not significant (*P* = 0.06). Significantly more infected squirrels were trapped in 2013 than in 2012 (OR = 4.45 [95% CI, 1.40 to 14.12]). Inclusion of a sine wave to represent the seasonal probability of infection was strongly supported in the model (ΔAIC = 6.4). The predicted monthly prevalence shows a strong seasonal signal, with a peak of infection occurring in early June (Fig. 4). The presence of ticks on a squirrel carcass increased the probability of infection (OR = 2.73 [95% CI, 1.38 to 5.34]).

**TABLE 3:** Summary table of results from a binomial generalized linear mixed model testing for B. burgdorferi *sensu lato* infection in gray squirrels

Fixed effect	Mean (estimated)	SE	*P* value	ΔAIC^*a*^
Intercept	−4.11	0.91	<0.001	
Yr (2013 vs. 2012)	1.49	0.57	0.010	+5.90
Age (subadult vs. adult)	0.61	0.33	0.063	+1.38
Tick (presence vs. absence)	1.00	0.34	0.003	+6.96

a ΔAIC, the change in AIC after removing each variable from the best-fit model.

## DISCUSSION

Previous studies have shown that invasive rodent species can be epidemiologically important in the maintenance of B. burgdorferi sensu lato and host-specific strains of the pathogen (8, 31). In this study, we assessed the potential role of invasive gray squirrels in the ecology of B. burgdorferi sensu lato in the United Kingdom. Our results suggest that invasive gray squirrels could be a potentially significant reservoir host, and further studies are required to quantify the role of the species in community disease dynamics. Also, many of our findings contrast with those reported from other invasive rodent species and challenge current views on the host restriction of B. burgdorferi sensu lato.

### Gray squirrels as tick hosts.

Gray squirrels in Scotland were found to be frequently parasitized with immature life stages of I. ricinus and were rarely parasitized with adult ticks, consistent with a previous study (36). Tick burdens were highly aggregated, as described previously for gray squirrels and ticks (36). This study supported previous findings that squirrels are important hosts of nymphs (36). This life stage represented 20% of the total ticks removed from squirrels compared to 1% of ticks removed from small mammals trapped at several field sites in Scotland (48). As a result, squirrels are more frequently exposed to B. burgdorferi sensu lato via feeding nymphs, as unfed larvae are rarely infected (61).

### Gray squirrels are infected with diverse genotypes of B. burgdorferi sensu lato.

Gray squirrels were frequently infected with a diverse range of B. burgdorferi sensu lato genotypes. This is consistent with previous research findings of high infection rates in sciurid species, both in their native ranges (62–64) and as introduced species (8). All genospecies previously known to occur in questing ticks in Scotland (20) were detected in the ear tissue of squirrels in this study, with evidence for transmission of B. afzelii and B. garinii from squirrels to feeding I. ricinus larvae. Infection with a high diversity of B. burgdorferi sensu lato genospecies has been previously reported in invasive Siberian chipmunks in France (9). The chipmunks in that study were also parasitized with a high proportion of nymphs, as were gray squirrels in this study, so both species are likely to be frequently exposed to B. burgdorferi sensu lato. We found a relatively low frequency of transmission of B. burgdorferi sensu lato genospecies to larvae feeding on infected gray squirrels. However, as most larvae had not completed the blood meal when removed from the squirrel carcass, the frequency of transmission may be underestimated. A captive xenodiagnosis study (65) of gray squirrels would be the most suitable approach to establish their reservoir competence for different B. burgdorferi sensu lato genospecies, with measurement of infection rates in engorged larvae that are allowed to molt into nymphs.

In contrast to the findings for invasive chipmunks in France (31), no evidence of host specificity within the two most frequent B. burgdorferi sensu lato genospecies infecting gray squirrels (B. afzelii and B. garinii) was found. Two of the three B. afzelii *clpA* alleles that we detected in gray squirrels had been previously recorded in small mammals from Scotland (48), suggesting that different host species may share some strains of B. afzelii. However, further strain typing would need to be carried out to confirm this. Our finding of significant phylogenetic clustering of squirrel B. afzelii *clpA* alleles among those found in questing ticks in Scotland is most likely to reflect incomplete spatial sampling of hosts (B. afzelii-infected squirrels were detected in only three regions in Scotland) rather than host specificity. Evidence of significant spatial structure was found in gray squirrel B. afzelii *clpA* alleles (see Table S3 in the supplemental material). Geographic structuring of B. afzelii has been reported previously and is thought to result from the limited movement patterns of the rodent reservoir hosts (51, 66). The spatial scale of sampling may be an important consideration in detecting patterns of host specificity of B. burgdorferi sensu lato strains among the vertebrate host community. The broad spatial scale of our study may not have been able to detect this effect. Indeed, the previous study, which found host-associated strains of B. burgdorferi sensu lato in invasive chipmunks in France, was conducted in a single forest (31). Data from further studies of gray squirrels from a smaller geographic area with comparative data from other reservoir hosts could be collected to investigate this further.

The high prevalence of B. garinii infections in gray squirrel ear tissues and its presence in internal tissues and transmission to feeding larvae were unexpected, as the genospecies is normally associated with avian hosts (25, 67). The emergence of a rodent-adapted ecotype of B. garinii has been documented previously in Europe and is thought to represent a complete host switch, with the loss of birds as transmission hosts (68). Here, we found no evidence for a gray-squirrel-adapted ecotype of B. garinii. Instead, phylogenetic analysis indicated that gray squirrels may be susceptible to any B. garinii strains circulating in the United Kingdom.

The host associations of particular genospecies are considered to be mediated by differential sensitivity of each genospecies to host complement (69, 70). Complement-mediated lysis of B. burgdorferi sensu lato is postulated to occur in the midgut of the tick during the blood meal and blocks onward transmission to the host (70). Despite a wealth of data supporting specific host associations of some B. burgdorferi sensu lato genospecies, there are a number of reports of host infection with unexpected genospecies. They include previous reports of B. garinii infections in rodents (23, 62, 71). Birds have also been shown to transmit the normally rodent-associated B. afzelii, but at a lower frequency and with shorter duration than the bird-associated genospecies B. garinii and B. valaisiana (72). Blood meal analysis studies, which use PCR to detect the remnants of a blood meal from a vertebrate host, as well as B. burgdorferi sensu lato infection (73), have reported a consistent minority of blood meals with results contrary to the accepted host associations (74). Collectively, these results suggest that there may be some permeability in the host barrier provided by host complement. This could be a result of biological variation in the bacterial complement regulator acquiring surface proteins (CRASPs) or due to heterogeneity in the host immune response (75). It also suggests that host-adapted B. burgdorferi sensu lato genospecies infecting vertebrate species outside the usual host range generally have reduced fitness. Such heterogeneity may play an evolutionary role and allow B. burgdorferi sensu lato to adapt to changes in the vertebrate host community.

In contrast to these previous studies, where non-host-associated genospecies were usually detected at lower frequencies, B. garinii was the most common genospecies infecting gray squirrels in this study. As gray squirrels seem to be susceptible to infection with any of the B. garinii genotypes circulating in Scotland, altered host defenses may be responsible for the different pattern of infection seen here. It has been proposed that invasive species may be under selection pressure to reallocate resources away from immune defenses toward other traits, such as reproduction, while their populations are expanding (76). This has been speculated to lead to reduced resistance to parasites and, possibly, increased susceptibility to novel parasites (76). In this case, lowered complement levels in the gray squirrel could allow increased transmission of B. garinii from infected ticks during a blood meal. Coinfections with other parasites may be an alternate potential mechanism for lowered complement levels in the species. Alternatively, gray squirrels may lack the ability to control B. garinii infection, because the genospecies is not found in the species' native range in North America. In comparison, B. garinii does cooccur in the native range of Siberian chipmunks and was present in a small proportion of infections from studies in its introduced range in France (9). Future comparative studies of immune function and parasite infections in invasive species are needed to discriminate between these hypotheses.

### Disseminated infection and transmission to tick larvae.

Infections with B. garinii and B. afzelii were found in the heart and bladder, demonstrating that both these genospecies are capable of establishing disseminated infections in gray squirrels. Testing ear biopsy samples for B. burgdorferi sensu lato appears to be a sensitive method of detecting infections in gray squirrels in comparison to testing internal tissues, as has been found previously for other rodent species (18).

We found that some gray squirrels that had negative ear biopsy results carried B. burgdorferi sensu lato-positive larvae. This could result from cofeeding transmission or undetected systemic infection of the gray squirrel. Cofeeding transmission is considered to be the most likely possibility, as no disseminated infections were detected in squirrels that tested negative on ear biopsy. However, we cannot confirm this, as the relative positions of feeding larvae and nymphs on the squirrel were not recorded and nymphs were not tested for infection.

### Host and environmental factors determining B. burgdorferi sensu lato prevalence in squirrels.

A strong seasonal pattern of B. burgdorferi sensu lato infection was seen in gray squirrels, with a predicted peak of infection occurring in early June. The peak in the predicted B. burgdorferi sensu lato prevalence lasts for approximately a month and then declines, suggesting that infection may not be life-long in the gray squirrel. In contrast to the results of our study, other species of rodents show life-long infection with B. burgdorferi sensu lato (65). We also found a tendency for subadult gray squirrels to be more likely to be infected than adults. Independent foraging of immature squirrels from the first breeding cycle occurs from March onward (38, 77) and coincides with the seasonal activity patterns of immature stages of I. ricinus, potentially driving high infection rates in subadults. Further longitudinal studies are needed to measure the duration of infection in gray squirrels.

### Conclusion.

Invasive gray squirrels were frequently infected with a variety of B. burgdorferi sensu lato genotypes. Our results suggest that gray squirrels may act as spillover hosts (6, 7) for B. burgdorferi sensu lato genotypes that are circulating locally in the native host community. Studies to measure the reservoir competence of the gray squirrel for strains of B. garinii and B. afzelii are needed to quantify the species role in community disease dynamics. Community effects may be complex, as invasive gray squirrels are also linked to the decline of another competent host for B. burgdorferi sensu lato, the red squirrel (33, 62, 63). Further studies to investigate how gray squirrels influence the abundance and competence of the host community for B. burgdorferi sensu lato are thus warranted. The results of the study also support the idea that invasive species may respond differently to endemic pathogens than native species. Infection of gray squirrels was most frequent with diverse genotypes of the normally bird-associated B. garinii, to which rodent species are usually resistant. Further comparative studies investigating complement levels in invasive gray squirrels and other species could investigate the hypothesis that reduced investment in innate immune defenses may be driving this altered pattern of infection with B. burgdorferi sensu lato.

## Supplementary Material

Supplemental material
