# Supplementary material for: An Invasive Mammal (the Gray Squirrel, Sciurus carolinensis) Commonly Hosts Diverse and Atypical Genotypes of the Zoonotic Pathogen Borrelia burgdorferi Sensu Lato
Source: Appl Environ Microbiol. 2015 Jun 4;81(13):4236–45. doi: 10.1128/AEM.00109-15 (PMC4475893; doi:10.1128/AEM.00109-15)
Supplement: Supplemental material [file supp_81_13_4236__index.html]

Supplemental material 

# An invasive mammal (grey squirrel, *Sciurus carolinensis*) commonly hosts diverse and atypical genotypes of the zoonotic pathogen *Borrelia burgdorferi* sensu lato.

## Supplemental material

- Supplemental file 1 -

  Plot of rarefaction curves for *Borrelia garinii clpA* allelic richness for questing *Ixodes ricinus* ticks sampled in continental Europe, England, and Scotland and gray squirrels (*Sciurus carolinensis*) from Scotland and northern England (Fig. S1); maximum-likelihood (ML) phylogeny of *ospC* alleles from *B. garinii*-infected gray squirrels (*S. carolinensis*)from Scotland and questing ticks (*I. ricinus*) from this study and from *I. ricinus* ticks sampled in a separate, unpublished study from Scotland (Fig. S2); ML phylogeny of *ospC* alleles from *Borrelia afzelli*-infected gray squirrels (*S. carolinensis*) from Scotland in this study and from questing ticks (*I. ricinus*) sampled from Scotland in a separate, unpublished study (Fig. S3); map of gray squirrel (*S. carolinensis*) sampling sites in Scotland and northern England (Fig. S4); positions of test sites in the 5S-23S *Borrelia burgdorferi sensu lato* intergenic spacer region used to discriminate between genospecies (Fig. S5); testing for phylogenetic clustering of *B. garinii ospC* (Table S1) alleles by host species in which the allele was detected, using three test statistics (Table S1); testing for phylogenetic clustering of *B. afzelli clpA* alleles by the host species in which the allele was detected (Table S2) and by the region where the gray squirrel was trapped (Table S3), using three test statistics; and testing for phylogenetic clustering of *B. afzelli ospC* alleles by sampled, using three test statistics (Table S4).

  PDF, 2.2M
