# Supplementary material for: An Invasive Mammal (the Gray Squirrel, Sciurus carolinensis) Commonly Hosts Diverse and Atypical Genotypes of the Zoonotic Pathogen Borrelia burgdorferi Sensu Lato
Source: Appl Environ Microbiol. 2015 Jun 4;81(13):4236–45. doi: 10.1128/AEM.00109-15 (PMC4475893; doi:10.1128/AEM.00109-15)
Supplement: Supplemental material [file AEM.00109-15_zam999116339so1.pdf]

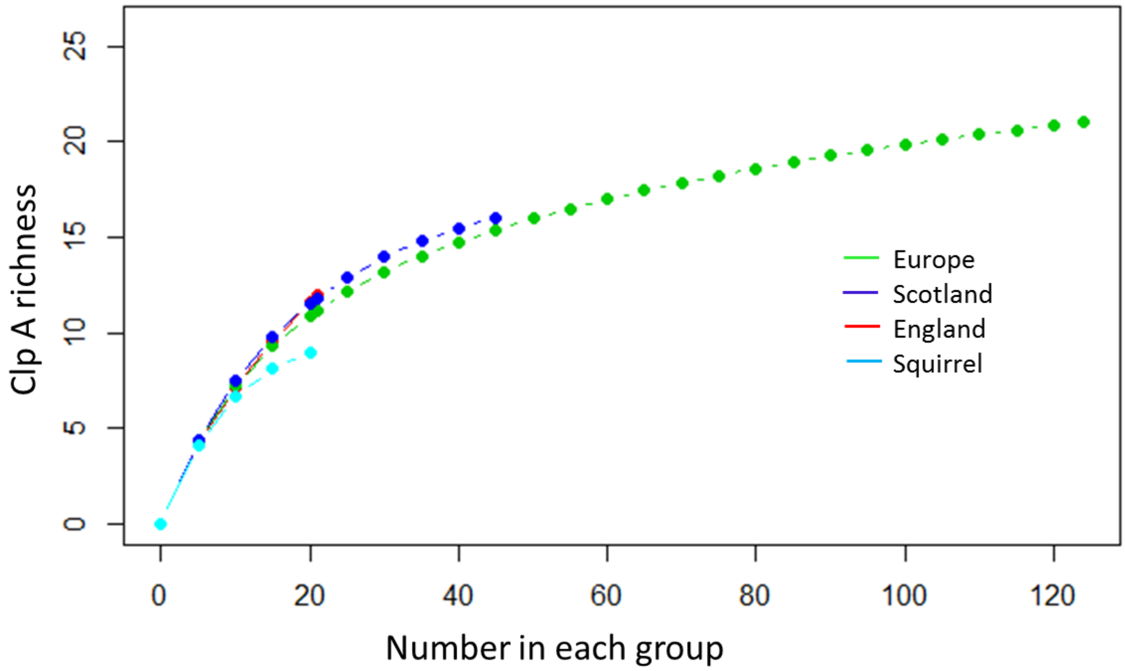

FIG S1: Plot of rarefaction curves for *Borrelia garinii* Clp A allelic richness for questing *Ixodes ricinus* sampled in continental Europe, England, Scotland and grey squirrels (*Sciurus carolinensis*) from Scotland and northern England. *B. garinii* Clp A allele numbers and frequencies from *I. ricinus* from Europe (n=58) and England (n=21) were retrieved from the *Borrelia* MLST website (1), alleles for Scotland (n=30) were from a previous PhD thesis (2) and also from this study, grey squirrel samples are from this study (n=20).

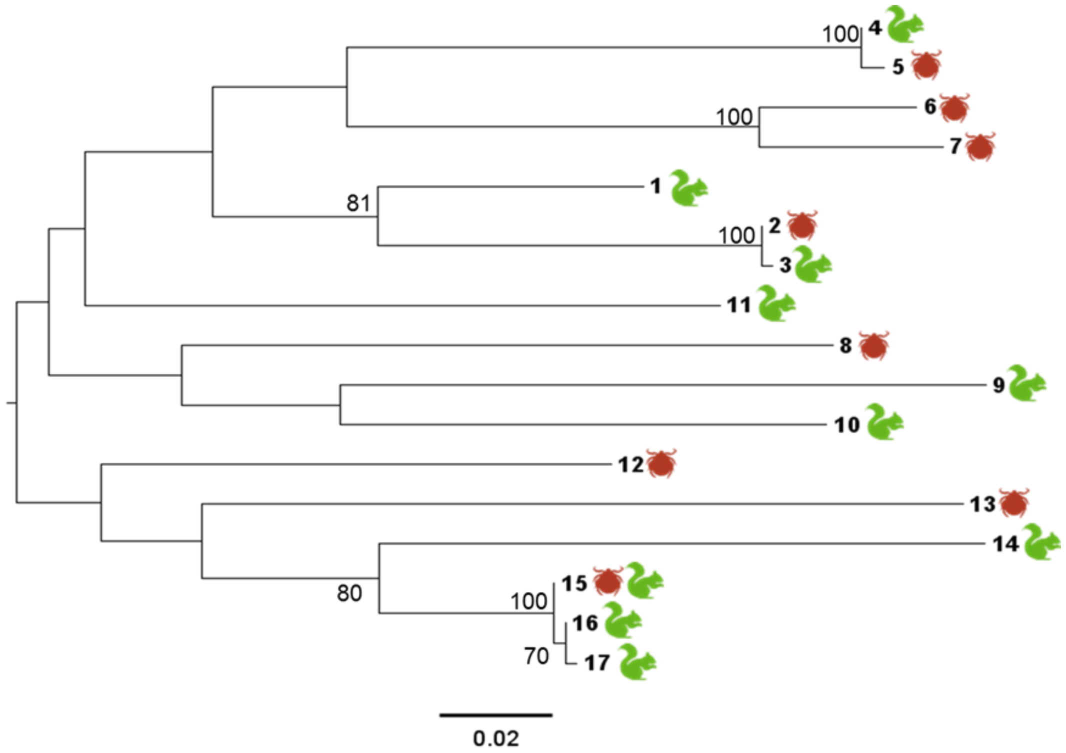

FIG S2: Maximum Likelihood (ML) phylogeny of Osp C alleles from *Borrelia garinii* infected grey squirrels (*Sciurus carolinensis*) (n=19) from Scotland and questing ticks (*Ixodes ricinus*) (n=9) from this study and from *I. ricinus* sampled in a separate unpublished study from Scotland (Millins unpublished). Each number on the tips of the tree represents a different allele, numbering of alleles is according to the author; a different allele number was assigned to sequences differing by one or more nucleotides. Ten Osp C alleles were detected in grey squirrels, allele 1 (n=4), allele 10 (n=3), allele 11, 14, 15 and 16 (all n=2), alleles 3, 4, 9, and 17 (all n=1). The ML tree was estimated based on the General Time Reversible model (3) in MEGA 6 (4) with 500 boot straps. A discrete Gamma distribution was used to model substitution rate differences among sites (5 categories (+G, parameter = 0.4633)). Only bootstrap values greater than 70 are displayed.

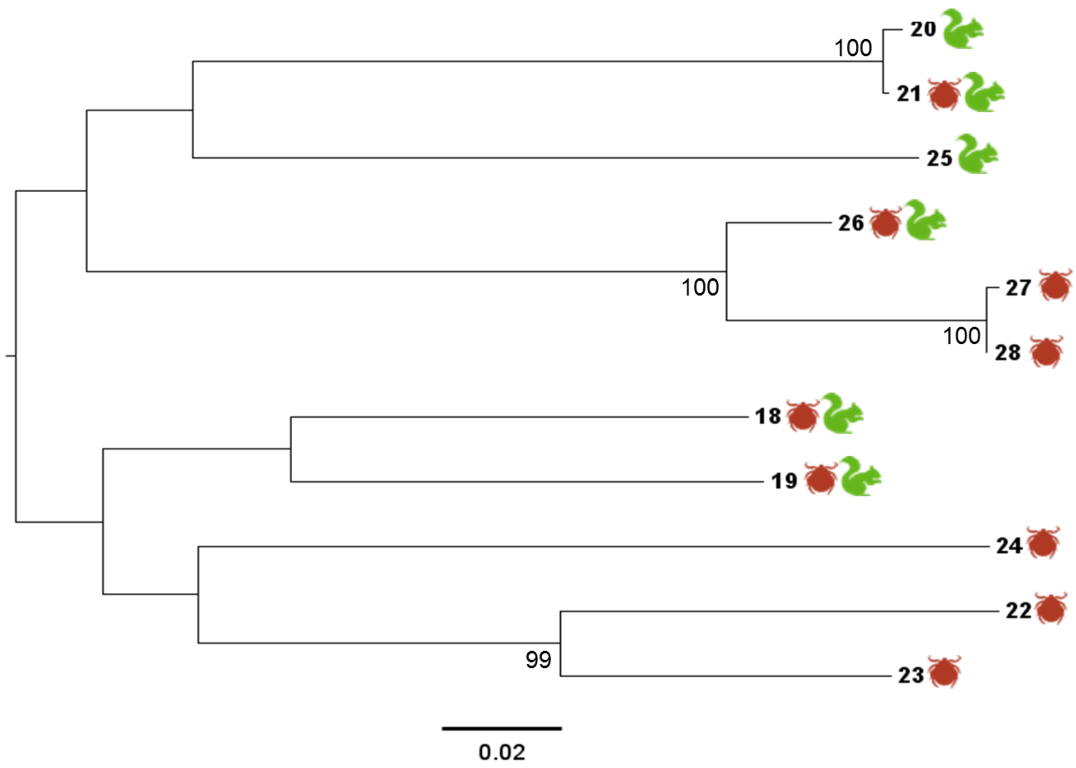

FIG S3: Maximum Likelihood (ML) phylogeny of Osp C alleles from *Borrelia afzelli* infected grey squirrels (*Sciurus carolinensis*) from Scotland in this study (n=12) and from questing ticks (*Ixodes ricinus*) sampled from Scotland (n=20) in a separate study (Millins unpublished). Each number on the tips of the tree represents a different allele, numbering of alleles is according to the author; a different allele number was assigned to sequences differing by one or more nucleotides. A total of six Osp C alleles were detected in grey squirrels: allele 18 (n=4), 19 (n=2), 20 (n=1), 21 (n=1), 25 (n=2), 26 (n=2). The ML tree was estimated based on the General Time Reversible model (3) in MEGA 6 (4) with 500 bootstraps. A discrete Gamma distribution was used to model substitution rate differences among sites (5 categories (+G, parameter = 0.5087)). Only bootstrap values greater than 70 are displayed.

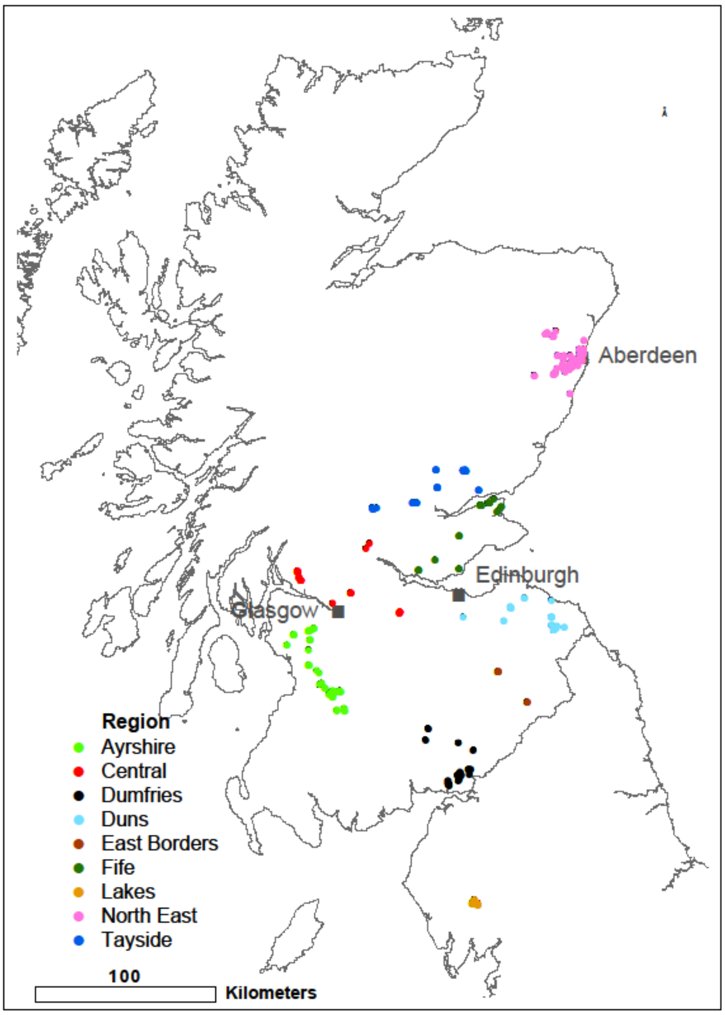

FIG S4: Map of grey squirrel (*Sciurus carolinensis*) sampling sites in Scotland and Northern England coloured by the control region squirrels were trapped from. Great Britain, Scotland outlines © Crown Copyright and Database Right 2011. Ordnance Survey (Digimap Licence). Map drawn in ArcMap 10 (Esri, California, USA).

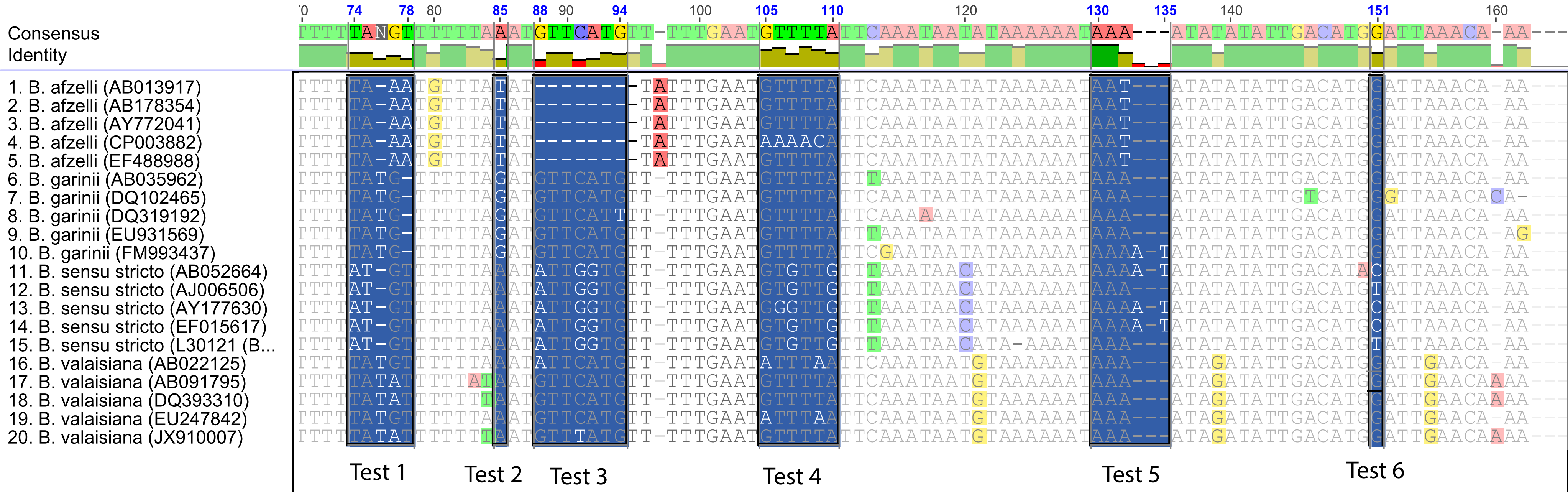

Figure S5: Position of test sites in the 5S-23S Borrelia burgdorferi sensu lato intergenic spacer region used to discriminate between genospecies. See Table S1 for recorded sequence polymorphisms at each test site.

TABLE S1 Testing for phylogenetic clustering of *Borrelia garinii* Osp C alleles by host species the allele was detected in (based on ML tree shown in Figure S2), using three test statistics described previously (5). All three test statistics test the null hypothesis that the sampled species are distributed randomly among tip nodes.

**Statistic**	**Observed mean (95% CI)**	**Null mean (95% CI)**	**p value**
Association Index[a]	0.8 (0.6-1.1)	1.4 (0.9-1.9)	0.04
Parsimony Score[b]	7.8 (7-8)	7.8 (6.3-8.9)	0.61
Monophyletic clade size[c] (Tick n=9)	2 (2-2)	1.8 (1.1 -3)	0.46
Monophyletic clade size (Squirrel n=19)	4.1 (4-5)	3.8 (2.1-6.6)	0.35

[a] Association Index, a measure of imbalance of internal nodes

[b] Parsimony Score, a number of state changes in phylogeny

[c] Monophyletic clade size, a measure of the clade size sharing the same trait at the tips.

TABLE S2 Testing for phylogenetic clustering of *Borrelia afzelli* Clp A alleles by the host species the allele was detected in (see Maximum Likelihood tree shown in FIG 3) using three test statistics previously described (5). All three test statistics tested the null hypothesis that the sampled species are distributed randomly among tip nodes.

Statistic	Observed mean (95% CI)	Null mean (95% CI)	p value
Association Index[a]	1.2 (0.6-1.9)	1.5 (1.3-1.6)	0.01
Parsimony Score[b]	8.9 (7-10)	10 (9.1-10.4)	0.02
Monophyletic Clade Score[c] (Grey Squirrel n=11)	2 (1-3)	1.6 (1.4 -2)	0.06
Monophyletic Clade Score (Tick n=32)	7 (6-10)	5.6 (4.2-7.4)	0.4

[a] Association Index, a measure of imbalance of internal nodes

[b] Parsimony Score, a number of state changes in phylogeny

[c] Monophyletic clade size, a measure of the clade size sharing the same trait at the tips.

TABLE S3 Testing for phylogenetic clustering of *Borrelia afzelli* Clp A alleles by region the grey squirrel (*Sciurus carolinensis*) was trapped from using three test statistics previously described (5). All three statistics test the null hypothesis that regions are distributed randomly among tip nodes.

Statistic	Observed mean (95% CI)	Null mean (95% CI)	p value
Association Index[a]	0.3 (0.1-0.7)	0.8(0.6-1)	0
Parsimony Score[b]	2.98 (3-3)	4.6 (3.7 -5)	0.01
Monophyletic Clade Size[c] (Fife n=2)	1(1-1)	1.1 (1-1.2)	1
Monophyletic Clade Size (Northeast n=6)	4.3 (4-6)	2 (1.5-4)	0.07
Monophyletic Clade Size (Tayside n=3)	1.8 (1-3)	1.2(1-1.8)	0.01

[a] Association Index, a measure of imbalance of internal nodes

[b] Parsimony Score, a number of state changes in phylogeny

[c] Monophyletic clade size, a measure of the clade size sharing the same trait at the tips.

TABLE S4: Testing for phylogenetic clustering of *Borrelia afzelli* Osp C alleles from by sampled species (see Maximum Likelihood tree, FIG S3) using three test statistics previously described (5). All three test statistics test the null hypothesis that sampled species are randomly distributed among tip nodes.

**Statistic**	**Observed mean (95% CI)**	**Null mean (95% CI)**	**p value**
Association Index[a]	1.4 (0.9-1.9)	1.7 (1.2-2.2)	0.11
Parsimony Score[b]	9.4 (8-11)	9.8 (8.5-11)	0.16
Monophyletic Clade Size[c] (Tick n=20)	3.8 (3-5)	3.6 (2.8-6)	0.15
Monophyletic Clade Size (Squirrel n=12)	2 (2-2)	2.1 (1.7-3)	0.6

[a] Association Index, a measure of imbalance of internal nodes

[b] Parsimony Score, a number of state changes in phylogeny

[c] Monophyletic clade size, a measure of the clade size sharing the same trait at the tips.

References

1. **Margos G, Vitorino L, Gatewood A, Hanincova K, Kurtenbach K**. 2014. *Borrelia burgdorferi* MLST.

2. **James MC**. 2010. The ecology, genetic diversity and epidemiology of Lyme borreliosis in Scotland. University of Aberdeen, Aberdeen.

3. **Nei M, Kumar S**. 2000. Molecular Evolution and Phylogenetics. Oxford University Press, New York.

4. **Tamura K, Stecher G, Peterson D, Filipski A, Kumar S**. 2013. MEGA6: Molecular Evolutionary Genetics Analysis Version 6.0. Mol. Biol. Evol. **30**:2725–2729.

5. **Parker J, Rambaut A, Pybus OG**. 2008. Correlating viral phenotypes with phylogeny: accounting for phylogenetic uncertainty. Infect. Genet. Evol. **8**:239–46.
